# An Ecological, Participatory, Integral and Contextualized Model (EPIC Model) of Family-School Connection: A Preliminary Analysis

**DOI:** 10.3389/fpsyg.2020.563506

**Published:** 2020-10-22

**Authors:** Mahia Saracostti, José Antonio Lozano-Lozano, Horacio Miranda, Laura Lara, Diana Martella, Taly Reininger

**Affiliations:** ^1^Escuela de Trabajo Social, Facultad de Ciencias Sociales, Director of UNESCO Chair Childhood/Youth, Education and Society, Universidad de Valparaiso, Valparaíso, Chile; ^2^Escuela de Psicología, Instituto de Ciencias Biomédicas, Universidad Autonóma de Chile, Santiago, Chile; ^3^Núcleo Científico Tecnológico en Ciencias Sociales y Humanidades, University of La Frontera, Temuco, Chile; ^4^Escuela de Psicología, Facultad de Ciencias Sociales y Humanidades, Universidad Autonóma de Chile, Santiago, Chile; ^5^Departamento de Trabajo Social, Facultad de Ciencias Sociales, Universidad de Chile, Santiago, Chile

**Keywords:** family involvement, children’s socioemotional development, children’s cognitive and learning abilities, child well-being, family and school relation, evidence-based practice

## Abstract

There are several programs that aim to strengthen the bond between families and schools that have shown a positive impact on this relationship as well as its effectiveness in improving academic and socioemotional child indicators. Most of the studies in this area come from Anglo-Saxon countries while in Latin America research is still scarce. Thus, this study aims to assess the influence of implementing an Ecological, Participatory, Integral and Contextualized Family-School Collaboration Model (EPIC) on family involvement, social-emotional development, and cognitive test outcomes in children in elementary school. Three possible hypotheses have been considered: (1) The EPIC Family-School Collaboration Model will have a positive and significant influence on the level of family involvement; (2) The EPIC Family-School Collaboration Model will have a positive and significant influence on the results of some cognitive tests; and; (3) The EPIC Family-School Collaboration Model will have a positive and significant influence on child social-emotional development. The study included 171 students who attended second and third elementary grades in schools in Chile during 2017 and fourth and fifth grades during 2019. The children were between 7 and 12 years old (M = 8.17, *SD* = 0.98), during 2017 and between 9 and 14 years old (M = 9.88, *SD* = 0.99), during 2019. The results show that the EPIC Family-School Collaboration Model has a positive and significant influence on the level of home-based involvement, memory and attention and intrapersonal skills in the first cycle of elementary education.

## Introduction

Numerous researchers have endorsed the crucial role of family in the academic performance and in the development of socio-emotional and cognitive abilities of children (Jeynes, 2012; Castro et al., 2015; Ma et al., 2016; Chavkin, 2017; Garbacz et al., 2017).

Most of the studies in this area are predominantly from Anglo-Saxon countries while in Latin America (Baker et al., 2016; Garbacz et al., 2017; Garbarcz et al., 2019; Eichin and Volante, 2018) research is still scarce. Therefore, this study aims to assess the preliminary influence of implementing an Ecological, Participatory, Integral and Contextualized Family-School Collaboration Model (EPIC) in Chile on family involvement, socio-emotional development, and cognitive tests in children in the first cycle of elementary education.

In the Chilean educational system, parental and/or guardian participation in school is considered a key factor in educational policy (MINEDUC, 2017). Due to previous research indicating that the degree of family involvement in school processes is a critical element in children’s development and learning during the first school stage (Caspe et al., 2006; Galindo and Sheldon, 2012), the EPIC model was designed for implementation in the first cycle of elementary education (Lara and Saracostti, 2020).

The principles of the Family-School EPIC Collaboration Model are: (1) an Ecological Perspective where children are in a situated and contextualized way, thus including their significant environment, family dimensions and educational and social-cultural contexts is considered key (Frey and Dupper, 2005; Saracostti, 2013). (2) a Participatory Approach that understands higher levels of participation of key actors is necessary to implement the solution model. (3) an Integral approach that supposes the efficient use of the social-educational intervention resources present in the school and in the community under the logic of comprehensive continuous improvement. (4) Contextualized co-construction in which training, planning, and implementation of the model is co-constructed with schools and in dialog with the team of professionals and the educational community.

The EPIC Collaboration Model seeks to encompass the family-school relationship from a logic of improvement, including the following phases:

Diagnostic Phase that seeks to identify and understand the family-school relationship from the perspectives of various actors in the educational community. The diagnosis gives rise to the definition of priorities, ranking needs and determining relevant courses of action (Raczynski and Muñoz, 2007; Saracostti and Villalobos, 2015). Schools were accessed through contact with school climate coordinators who then provided access to the school management team. Once the family involvement surveys and cognitive and socioemotional development tests were applied, results were shared with school management and professional teams who provided further complementary school data. This information was then utilized in co-constructing the model based on scientific evidence that demonstrated family involvement had a positive impact on students’ learning processes (UNESCO, 2014).

1. Initial Training Phase seeks to install capacities within schools, based on the findings of systematic reviews and meta-analyses on the positive impacts of interventions on student behavior, school climate, academic achievement, and school retention (Wilson et al., 2011; Maynard et al., 2012). This phase is aimed at principals, management teams, teachers and psychosocial school professionals since the literature indicates the need to have the political will of the establishments, recommending actions be channeled from the governance of the schools as a key condition for developing effective plans that involve the family (Hoover-Dempsey and Sandler, 2005). This stage included (a) 2 days of training aimed at school educational teams: teachers, psychosocial professionals, and managers and, (b) A 1-day training session with the families of students included in the study. Both training activities lasted 8 h and included theoretical information on school family collaborations, the review of national and international experiences, and the design of a collaborative Family-School intervention plan for participating schools.
2. Intervention Phase that aims to accompany and advise the implementation of the Family-School Intervention Plan generated from and for each school, which is inserted within the Family-School Collaboration Model. A workshop was held with school and parent representatives in order to determine the strengths and limitations of family school relations. With this information an action plan was developed for each school considering the principles of the EPIC model. School psychosocial teams (psychologists and social workers) were in charge of implementing the plan in each school and were accompanied by members from the research team in monthly meetings. These workshops created a space for each school to develop new strategies to encourage family involvement and included a reception protocol for students and families, a parenting skills workshop, a citizenship fair and the design if a traveling notebook that arrived at the homes if students in other schools.
3. Systematization, Monitoring and Evaluation Phase which is designed in parallel to the intervention design process and which takes into consideration the specific context of each school as well as each intervention (Saracostti and Villalobos, 2015). Field notes were recorded in regards to the implementation processes of school plans including activities (description of what occurred during each school visit), ideas, reflections, doubts (including interpretations, as well as explanations of what was occurring) and next steps (changes regarding upcoming activities and/or interventions). Furthermore, a second wave of the standardized measures was undertaken during this phase with the family involvement survey applied during the last month of the EPIC model’s execution.

The EPIC model, consistent with its principles and application of its four phases, lasted 16 months and contributed to the comprehensiveness and networking between the diverse psychosocial programs being implemented in parallel within the schools. A facilitator from the research team was in charge of overseeing the implementation of the EPIC model by accompanying and guiding the process, working on average 4 h a week with school management teams and/or professionals as well as with families.

In this study, three possible hypotheses have been considered:

1. The EPIC Family-School Collaboration Model will have a positive and significant influence on the level of family involvement in the first cycle of elementary education, this being higher than that observed in the schools that make up the control group;
2. The EPIC Family-School Collaboration Model will have a positive and significant influence on the results of some cognitive tests of children in the first cycle of elementary education, this being higher than that observed in the schools that make up the control group; and,
3. The EPIC Family-School Collaboration Model will have a positive and significant influence on the social-emotional development of children in the first cycle of elementary education, this being higher than that observed in the schools that make up the control group.

## Methods

### Participants

The study included 171 students who attended second and third elementary grades in schools in three regions in Chile (O’Higgins, El Maule and La Araucanía) during 2017 and fourth and fifth grades during 2019. They were randomly assigned to the control group (106) and the experimental group (65) using non-probability purposive sampling (Kerlinger and Lee, 2002). The children had high levels of vulnerability according to the student vulnerability index issued by the Chilean Ministry of Education. The participants were evaluated at two moments, before the intervention (year 2017) and after the intervention (year 2019).

The children were between 7 and 12 years old (M = 8.17, *SD* = 0.98), during 2017 and between 9 and 14 years old (M = 9.88, *SD* = 0.99), during 2019. 44.6 and 55.6% were girls and boys, respectively.

This study was carried out in accordance with the ethical recommendations of the Chilean National Commission for Scientific and Technological Research. The protocol was approved by the Ethics Committee of the Universidad de La Frontera (Acta 066-2017, Folio 036-17). All the subjects provided written informed consent in accordance with the Declaration of Helsinki.

### Instruments

#### Assessment of Family Involvement

Hoover-Dempsey and Sandler’s Parental Involvement model and scales were used to evaluate parental involvement in children’s education. These were translated into Spanish and validated by a panel of experts in Chile (Reininger, 2014). The scales included the Parental Involvement forms scale (with two subscales: home based involvement, 5 items, and school based involvement, 5 items); teacher invitations for involvement scale (6 items); and the general school invitations scale (6 items). The fist scale has a four-point Likert response scale, from 1 (never) to 4 (always), while the rest was a 5-point scale Likert response, from 1 (strongly disagree) to 5 (strongly agree).

#### Evaluation of Learning Outcomes

Educational psychology battery EVALÚA (García and González, 2006), was used to assess learning outcomes and basic cognitive processes. The instrument has been validated and assessed in Chile. Three subtests of the battery were used in this study: Memory and Attention (MA) and two subtests of reasoning, Analogical Thinking (AT) and Perceptual Organization (PO). (1) MA is composed of 4 tasks and a total of 22 items; direct punctuation (DP) is calculated with the formulas: PDMA = ΣA-(E + O); (2) AT involves 2 tasks and a total of 20 items; PD = A-E/3 is the used to calculate the DP; and (3) PO is composed of 2 tasks and a total of 34 items and the DP of each part is calculated with the formula ΣA-E/31. In each subtest, the sum of the partial DPs corresponds to total DP. In the formula, A is the number of correct responses; E is the number of errors; O is the number of omissions, and the number 3 correspond to the number of alternatives minus 1.

#### Assessment of Socioemotional Development

The EQ-I: YV questionnaire (Emotional Intelligence Inventory: Young Version, Bar-On and Parker, 2000) adapted and validated in Spanish (Ferrándiz et al., 2012) evaluate social and emotional competences of children and adolescents. It is composed of 60 items grouped into 5 subscales: interpersonal, intrapersonal, adaptability, stress management, and general mood. The response scale ranged from 1 (rarely) to 4 (nearly always).

### Procedure

This study focusses on the effectiveness of interventions to strengthen the link between families and schools. The data referring to the students (evaluation of learning outcomes and assessment of socioemotional development) was collected during school hours and were registered in digital format on the schools’ computer rooms during three sessions. The data referring to the families (family involvement) were collected in paper format during parent teacher meetings.

### Analysis Plan

To compare the response between the experimental and control groups, the research employed a quasi-experimental pre-post longitudinal design (Hernández et al., 2006) with case study matching based on age, school size, classroom, and gender (Lukas and Santiago, 2004). This determined that no significant differences between the experimental and control group were present at the beginning of the study, as shown in Table 1.

**TABLE 1:** Baseline characteristics of students and parents.

Students and parents characteristics	Control (*N* = 106)	Intervention (*N* = 65)	*P*-value
Students’ gender			0.237
Girls	44 (41.5)	33 (50.8)	
Boys	62 (58.5)	32 (49.2)	
Student age			0.372
Mean (*SD*)	8.25	8.09	
Median (IQR)	8(1)	8(2)	
Min-max	7–12	7–11	
Student education level			0.656
Grade 2	64 (60.4)	37 (56.9)	
Grade 3	42 (39.6)	28 (43.1)	
Parent relationship with child			0.322
Mother	94 (88.7)	56 (86.2)	
Father	4 (3.8)	5 (7.7)	
Uncle or aunt	3 (2.8)	0 (0.0)	
Grandfather or grandmother	5 (4.7)	3 (4.6)	
Others	0 (0.0)	1 (1.5)	
Mother’s education			0.386
No studies	2 (1.9)	0 (0.0)	
Primary	18 (17.0)	18 (27.7)	
Secondary	71 (67.0)	40 (61.5)	
Vocational School	12 (11.3)	5 (7.7)	
Bachelor’s degree or higher	3 (2.8)	2(3.1)	
Father’s education			0.099
No studies	0 (0.0)	1(1.5)	
Primary	29 (27.4)	18 (27.7)	
Secondary	65 (61.3)	37 (56.9)	
Vocational School	11 (10.4)	4 (6.2)	
Bachelor’s degree or higher	1 (0.9)	5 (7.7)	

**Significant at P < 0.05. chi-square test. IQR, interquartile range.**

To verify that the comparisons of the main effects of the intervention, time, and interaction were not affected by a difference at the beginning of the study, we proceeded to compare the dependent variables in the initial time between the intervention group vs. the control. The results showed that most of the dependent variables did not present significant differences by the non-parametric Mann–Withey *U*-test. However, the variables analogical thinking and stress management did show significant differences before the intervention, and thus they were removed from the study. Subsequently, the ANOVA model was evaluated on the average ranking non-parametric transformation (Conover and Iman, 2012) with the main effects of the intervention vs. the control, the moments in time, and the interaction between the intervention and time. In this case, the interaction corresponds to a hypothesis test of the trend over time, with a null hypothesis of parallelism and an alternative hypothesis of non-parallelism that includes divergence, convergence, and trend crossing (Newrnan et al., 1999; Shadish et al., 2002). In some cases, the hypothesis of the interaction was not significant, but the main effects of the intervention or time did show significant differences, which in this study corresponds to divergence or crossing of trends where the distance between the confidence intervals increases significantly toward the end of the study.

The aim is to check whether introducing the set of activities that make up the intervention model (EPIC model) in the Experimental Group (Ge) causes differential effects in its post-treatment performance in relation to the Control Group (Gc). The groups formed are not entirely equivalent nor will the control exercised over the experimental conditions be absolute (Sarría-Santamera, 2020) although constant maintenance was used and subject to self-control. The use of different evaluation instruments is contemplated (cognitive tests and social-emotional development instruments in students, as well as family involvement in parents and/or guardians) in order to provide more extensive and complex information on the processes evaluated.

Because dependent variables did not have normal distribution, the ranking transformation was applied with tie correction to obtain a non-parametric analysis of the hypothesis tests, as proposed by Conover and Iman (2012). The effect assessment was performed through a general linear model applied on dependent variables with ranking transformation with tie correction, with independent variables of type dummy based on intervention (X1: 0-Control/1-Experimental), time (X2: 0-Start time/1-end time) and the interaction between intervention and time (X1^∗^X2). Hypothesis tests were performed using the probability value of student’s *t*-distribution of the model parameters, equivalent to the probability value of the Snedecor F distribution with 1 and n-1 degrees of freedom from the ANOVA table, used as significance level 0.05.

## Results

The characteristics of students and their parents were collected in 2017. In terms of the age, gender, and level of education of the students, no significant differences were found between the intervention and the control group (*p* = 0.237, *p* = 0.372, and *p* = 0.656 respectively). As for the characteristics of the parents, no significant differences were found between the intervention and the control group in regards to the type of relationship with the child, and mother and father’s education (*p* = 0.322, *p* = 0.386, *p* = 0.09, respectively).

Regarding the first hypothesis, as shown in Table 2, the EPIC Family-School Collaboration Model had a positive and significant influence only on “*Home based involvement”* in the first cycle of primary education. The ANOVA showed significant main effects of *Group* [*F*_(1, 317)_ = 3.907; *p* = 0.048] but not over time [*F*_(1, 317)_ = 0.747; *p* = 0.0001] indicating better performance in the intervention group (mean = 12.00; *SD* = 2.46) in comparison to controls (mean = 11.71; *SD* = 2.95) and in post-intervention (mean = 12.20; *SD* = 2.67) however, after the intervention, the control group proved worse performance (mean = 10.93; *SD* = 3.29).

**TABLE 2:** EPIC family-school collaboration model on the level of *family involvement.*

Variable	EC	Ss	*F*-test	*P*-value
Home based	Intervention	33.091	3.907	**0.048**
involvement	Time	6.332	0.747	0.387
	Intervention*Time	7.661	0.904	0.342
School based	Intervention	9.232	1.161	0.282
involvement	Time	8.993	1.131	0.288
	Intervention*Time	0.823	0.104	0.748
Student invitations for	Intervention	6.941	0.657	0.418
involvement scale	Time	0.265	0.025	0.874
	Intervention*Time	6.469	0.612	0.434
Teacher invitations for	Intervention	53.265	2.322	0.129
involvement scale	Time	67.746	2.953	0.087
	Intervention*Time	41.069	1.790	0.182
General school	Intervention	10.563	0.580	0.447
invitations scale	Time	174.057	9.557	**0.002**
	Intervention*Time	15.493	0.851	0.357

**Bold values are significant at p < 0.05. EC, Experimental Condition; SS, Sum of Squares.**

*School based involvement* did not show significant main influence of the *Group* [*F*_(1, 317)_ = 1.161; *p* = 0.282] and *Time* [*F*_(1, 317)_ = 1.131; *p* = 0.288]. *Teacher invitations for involvement scale* did not show significant main influence of the *Group* [*F*_(1, 315)_ = 2.322; *p* = 0.129] and *Time* [*F*_(1, 315)_ = 2.953; *p* = 0.087]. *General school invitations scale* did not show significant main influence of the *Group* [*F*_(1, 316)_ = 0.580; *p* = 0.447] and *Time* [*F*_(1, 316)_ = 9.557; *p* = 0.002].

Regarding the second hypothesis, as shown in Table 3, the EPIC Family-School Collaboration Model had a positive and significant influence only on the level of *“memory and attention.”* The ANOVA showed significant main effects of *Group* [*F*_(1, 303)_ = 14.56; *p* = 0.0002] and *Time* [*F*_(1, 303)_ = 34.34; *p* = 0.0001] indicating better performance in the intervention group (mean = 21.09; *SD* = 19.92) in comparison to controls (mean = 16.05; *SD* = 18.35) and in post-intervention (mean = 35.94; *SD* = 15.93) in comparison to pre-intervention (mean = 25.30; *SD* = 16.24).

**TABLE 3:** EPIC family-school collaboration model on the level of *learning outcomes.*

Variable	EC	Ss	*F*-test	*P*-value
Memory and	Intervention	4436.249	14.563	**<0.001**
attention	Time	10463.580	34.349	**<0.001**
	Intervention*Time	565.061	1.8550	0.174
Perceptive	Intervention	16.669	0.2891	0.591
organization	Time	1683.785	29.2452	**<0.001**
	Intervention*Time	25.120	0.4363	0.509

**Bold values are significant at p < 0.001. EC, Experimental Condition; SS, Sum of Squares.**

*Organizational thinking* did not show significant main influence of the Group [*F*_(1, 301)_ = 0.239; *p* = 0.625) and *Time* [*F*_(1, 301)_ = 27.49; *p* = 0.0001].

Finally, regarding the third hypothesis, as shown in Table 4, the EPIC Family- School Collaboration Model had a positive and significant influence only on the level of *“intrapersonal skill.” The ANOVA* showed significant main effects of *Group* [*F*_(1, 333)_ = 10.6; *p* = 0.001] and *Time* [*F*_(1, 333)_ = 4.5; *p* = 0.03] indicating better performance in the intervention group (mean = 16.3; *SD* = 3.8) in comparison to controls (mean = 15.3; *SD* = 3.9) and in post-intervention (mean = 16.2; *SD* = 3.7) in comparison to pre-intervention (mean = 15.6; *SD* = 4.1).

**TABLE 4:** EPIC family-school collaboration model on the level of *socioemotional development.*

Variable	EC	Ss	*F*-test	*P*-value
Interpersonal skill	Intervention	89358.961	2.723	0.099
	Time	23865.985	0.727	0.394
	Intervention*Time	1277.589	0.038	0.843
Intrapersonal skill	Intervention	337811.28	10.555	**0.001**
	Time	144452.25	4.513	**0.034**
	Intervention*Time	86739.84	2.710	0.100
Adaptability	Intervention	19.697	0.534	0.465
	Time	22.684	0.615	0.433
	Intervention*Time	77.428	2.100	0.148
General mood	Intervention	27271.22	0.831	0.362
	Time	69235.78	2.11	0.146
	Intervention*Time	47.74	0.0015	0.969

**Bold values are significant at p < 0.001 or p < 0.05. EC, Experimental Condition; SS, Sum of Squares.**

“Adaptability” did not show significant main influence of the Group [*F*_(1, 333)_ = 0.534] and Time [*F*_(1, 333)_ = 0.615]. “*Interpersonal skill*” did not show significant main influence of the Group [*F*_(1, 333)_ = 2.72; *p* = 0.099] and *Time* [*F*_(1, 333)_ = 0.727; *p* = 0.394]. Stress management did not show significant main influence of the *Group* [*F*_(1, 333)_ = 0.8895; *p* = 0.346] and *Time* [*F*_(1, 333)_ = 0.2247; *p* = 0.635]. General mood did not show significant main influence of the *Group* [*F*_(1, 333)_ = 0.8311; *p* = 0.362) and *Time* [*F*_(1, 333)_ = 2.11; *p* = 0.146].

## Discussion

Family-school collaboration allows for a broader conceptualization of school and family roles, their relationships and the impact on the all-round development of children (Patrikakou et al., 2005; Christenson and Reschly, 2010; Yamauchi et al., 2017). From this perspective, families and schools are protagonists in the construction of their roles and forms of involvement, since they generate new and varied actions to relate, considering the specific context of each educational community. The positive effects of the family-school connection benefit all the actors involved, fostering a positive school climate (Cowan et al., 2002; Wyrick and Rudasill, 2009). The most significant impact is observed in the greater coherence and mutual support between the family and the school, becoming a protective factor for children and their families (Phelan et al., 1998).

Programs that seek to strengthen the relationship between families and schools are theoretically supported by an eco-systemic perspective which recognizes the importance of positive and fluid interactions between the different spheres of a child’s life. According to Bronfenbrenner and his ecological theory (1987), the interrelations between schools and families play an essential role in ensuring a child’s socioemotional and cognitive development. It is thus key that both spheres be considered in the development of comprehensive child protection systems. Consistent with the ecosystem perspective, Epstein (2013) overlapping of spheres of influence model was developed specifically in order to explain and guide research and intervention in the field of family-school relations. This model combines symbolic interactionism, Merton’s reference group theory and Elder’s life cycle thus including three relevant spheres that interact in children’s learning: the school, the community and the family.

As explained before, much has been published in Anglo Saxon journals in regard to psychosocial interventions within schools and their impacts on a wide range of areas including student behavior, school climate, academic achievement and school retention, among others. For example, the “WSCC” model from Atlanta (Lewallen et al., 2015) is a model similar to the EPIC model. This model addresses family school relations from a systemic, integrated, and collaborative approach to health and learning. It is designed to provide a framework for collaborative decision making involving multiple institutions found within schools in order to achieving greater alignment and integration of educational policies and programs as well as incorporating family and community participation, differentiating the role each plays in children’s learning and the potential of partnerships between families and schools.

Regarding the aspects of the EPIC model that are visible in the schools’ implementation, the idea of an integral approach and that of contextualized co-construction stand out. It was possible to observe that within participating schools, the EPIC model was implemented in an articulated manner with programs already operating in the schools. This articulation was led by directive teams and implemented by social workers and psychologists thus establishing coordination in regards to family interventions. Furthermore, the participatory methodology allowed for the identification of each school’s strengths, needs, and projections thus the action plans were pertinent to each schools’ and families’ contextual reality.

Regarding family involvement, the results of our study indicated that families who participated in the experimental group reported significantly higher rates of home involvement than the control group. These are positive findings since the EPIC model seeks to encompass the family-school relationship from an integral and ecological perspective. These results confirm similar findings to other studies in which family involvement at home can be stimulated and strengthened by different types of interventions (Bellei et al., 2002; Hoover-Dempsey and Sandler, 2005). This finding is particularly promising since home involvement in its different expressions (such as: *someone in this family (father, mother and/or guardian) helps the child study for test”* or “*someone in this family (father, mother and/or guardian) practices spelling, math or other skills with the child”)* may be more highly related to positive student outcomes than other more visible forms of parental school involvement (such as *“someone in this family attends parent–teacher association meetings*” or “*someone in this family attends special events at school*”).

Relating cognitive processes, one of the aims of this study was to evaluate the influence of the EPIC Family-School Collaboration Model on the results of some cognitive tests of attention and memory, considered as predictors of academic trajectories, in particular for the early elementary grades, (i.e., Stipek et al., 2015). Results of our study indicated better performance of children participating in the intervention group in comparison to the control group, partially confirming a positive and significant influence of EPIC on cognitive processes and academic achievement (i.e., Fan and Chen, 2001). Another important result is the improvement over time of cognitive functions evaluated, irrespective the intervention, in line with neurocognitive studies (i.e., Nagy et al., 2004).

Finally, we found the intervention had a positive and significative influence over intrapersonal skill (ability to understand one’s own emotions and communicate them to others). Literature has highlighted the results of the positive influence of family involvement in school over the socioemotional development of children (Garbacz et al., 2017). Although available literature is scarcer regarding the effectivity of the interventions over socioemotional outcomes in comparison with academic ones, there is also a robust body of studies in concordance with the results founded in our study. For example, in a recent meta-analysis of the effects of family-school interventions on children’s social-emotional functioning (Sheridan et al., 2019) conclude that these effects are positive and significant when analyzing data from 117 different studies. In a similar way, in another recent meta-analysis of the effects of family-school partnership interventions on academic and social-emotional functioning (Smith et al., 2019) the results are concordant.

One of the main weaknesses is that the study utilized a thematic or convenience sample, furthermore the sample size was small. Therefore, one of the main challenges for future research in Chile and Latin America is the need for studies with probabilistic and larger samples. On the other hand, the time factor may have possibly hindered the possibility of reaching more decisive results considering that establishing trusting professional relationships between schools, families, and the research teams take time and are key in these types of interventions. Therefore, we suggest the need to undertake quasi-experimental designs of a greater time scope.

## Data Availability Statement

The raw data supporting the conclusions of this article will be made available by the authors, without undue reservation.

## Ethics Statement

The studies involving human participants were reviewed and approved by the Ethics Committee of the Universidad de La Frontera (Acta 066-2017, Folio 036-17). Written informed consent to participate in this study was provided by the participants’ legal guardian/next of kin.

## Author Contributions

MS developed the study concept, the study design and drafted the manuscript. DM, TR, and LL performed the data collection. HM and JL-L performed the data analysis and interpretation under the supervision of MS. DM, TR, LL, HM, and JL-L substantially contributed to the interpretation of the data and provided important critical revisions. All authors approved the final version of the manuscript and also agreed to be accountable for all aspects of the work.

## Conflict of Interest

The authors declare that the research was conducted in the absence of any commercial or financial relationships that could be construed as a potential conflict of interest.
